# Computational design and evaluation of multiepitope vaccines against herpes simplex virus type 1

**DOI:** 10.3389/fimmu.2025.1581571

**Published:** 2025-06-04

**Authors:** Zibo Zhao, Weixiong Wang, Jiaping Pang, Bei Zhou, Xin Li, Yifei Wang, Kai Zheng, Zhe Ren

**Affiliations:** ^1^ Institute of Biomedicine, College of Life Science and Technology, Jinan University, Guangzhou, China; ^2^ Guangdong Province Key Laboratory of Bioengineering Medicine, Jinan University, Guangzhou, China; ^3^ The Key Laboratory of Virology of Guangdong, Jinan University, Guangzhou, China; ^4^ School of Pharmacy, Shenzhen University Medical School, Shenzhen University, Shenzhen, China

**Keywords:** HSV-1, multiepitope vaccine, molecular dynamics simulation, molecular docking, immune response

## Abstract

**Introduction:**

Herpes simplex virus type 1 (HSV-1) is a prevalent human pathogen, causing infections in various tissues and leading to severe complications such as herpes simplex encephalitis and cognitive impairments. Despite existing antiviral treatments, recurrent infections and the lack of effective vaccines highlight the need for new preventive measures.

**Methods:**

We employed immunogenomic and bioinformatics methods to design two multi-epitope vaccine constructs 1 and 2 against HSV-1. The Immune Epitope Database was used to identify B-cell and T-cell epitopes from HSV-1 glycoproteins. The IFN epitope server and the IL4pred/IL-10pred server were used to ascertain the activation possibility of IFN-γ, IL-4, and IL-10. The NetMHC-4.0 and NetMHCII2.3 servers were used to identify MHC epitopes. The constructed vaccine was analyzed for antigenicity and allergenicity using the VaxiJen v2.0 and AllergenFP servers. The three-dimensional structure of the vaccine construct was constructed using the AlphaFold3 tool. The ClusPro 2.0 server was utilized for molecular docking and the Desmond module in Schrodinger 2021-1 was utilized for molecular dynamics and MM/PBSA analysis. The immunogenicity and the corresponding immune response curves were analyzed using the C-ImmSim server.

**Results:**

Bioinformatics analysis demonstrated that these vaccines exhibited both good affinity and immunogenicity, and were non-toxic and non-allergenic to the host. In addition, vaccine construct 2 exhibits superior stability and binding affinity with TLR9, and is more effective in triggering a robust immune response.

**Discussion:**

This approach targets the key mechanisms of HSV-1 entry and TLR-mediated immune responses, providing a potential strategy for preventing and treating HSV-1 infections. Furthermore, the identified and optimized vaccine construct offers a promising avenue for developing a preventive vaccine against HSV-1, addressing the critical need for better control of this widespread virus.

## 1 Introduction

Herpes Simplex Virus Type 1 (HSV-1) is a widespread human pathogen (1), with seropositivity rates reported to range from 45% to 98% globally (2, 3). It can cause infections in the lips, eyes, or genitals (4, 5). After an initial infection in epithelial cells, HSV-1 becomes latent in neurons of the peripheral nervous system and can reactivate periodically (6, 7). The virus can also reach the central nervous system, where its replication in the brain may lead to herpes simplex encephalitis (8, 9). Recurrent reactivation of HSV-1 is a significant pathogenic mechanism underlying amnestic mild cognitive impairment (aMCI), Alzheimer’s disease and Parkinson’s disease (10–13). This causes substantial harm to patients, severely impacting their daily lives and overall health. Antiviral drugs such as idoxuridine and acyclovir are used to treat HSV-1 infections by incorporating into the replicating viral DNA and exerting antiviral effects (14, 15). Although these drugs can reduce and alleviate symptoms following infection, they still possess significant side effects that are difficult to eliminate. Currently, vaccines are of crucial importance in preventing the infection and transmission of the virus (16). However, there are no clinically approved vaccines for the prevention or treatment of HSV-1 infections. The serious consequences of recurrent HSV-1 infections pose a major threat to public health, underscoring the urgent need for the development of new preventive measures against HSV-1 infection.

HSV-1 enters host cells through interactions between its envelope glycoproteins (gB, gC, gD, and the gH/gL complex) and receptor-associated proteins on target cells (17). Typically, gD binds to host receptors, which activates gH/gL and converts pre-fusion gB into its active form, facilitating membrane fusion (18). Toll-like receptors (TLRs) have been shown to play a crucial role in the early defense against viruses by recognizing viral components and activating innate immune signaling pathways (19). This leads to the induction of IFN-1, pro-inflammatory factors, cytokines, and chemokines (20). The recognition of viral proteins by membrane receptors and viral nucleic acids by endosomal TLRs is vital for controlling HSV-1 infection (21). The TLR family proteins have been shown to participate in HSV-1 recognition and IFN induction during viral entry and replication (22–25). Consequently, hindering this process can impede HSV-1 from entering host cells, thus providing a potential strategy for the prevention and treatment of HSV-1 infections. By addressing the key mechanisms of HSV-1 entry and TLR-mediated immune responses, this approach highlights the critical points for therapeutic intervention in managing HSV-1 infections.

**Figure 1:**
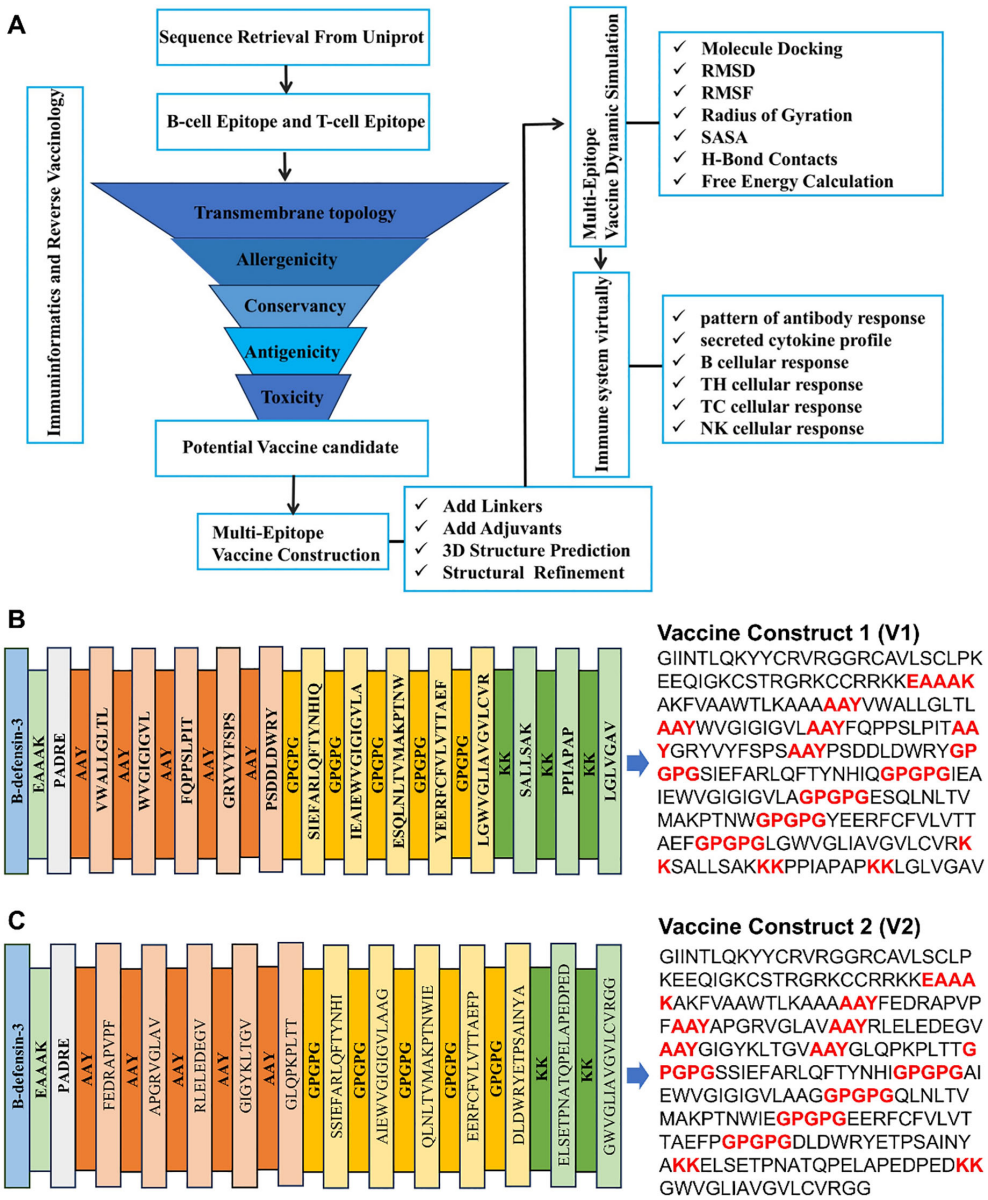
Vaccine construction. **(A)** Schematic diagram of vaccine construct. **(B, C)** Amino acid sequence of vaccine construction V1 and V2.

In this study, a series of immunogenomic and bioinformatics methods were employed to identify a number of B-cell and T-cell epitopes from HSV-1 glycoproteins that exhibited favorable immune parameters (**Figure 1A**). These epitopes have the capacity to induce immune-related cytokines, thereby contributing to the vaccine’s robust immunogenicity, as well as its non-allergenic and non-toxic properties. By linking these epitopes with specific linkers, we constructed multi-epitope vaccine constructs 1 and 2. Finally, we conducted a series of analyses and evaluations on the constructed vaccine, which further indicated that vaccine construct 2 is more suitable for triggering a robust immune response.

## 2 Materials and methods

### 2.1 Biophysical properties analysis

The envelope glycoproteins B, C, D, H, and L of HSV-1 were retrieved in FASTA format from the UniProt database. The antigenic properties of the selected viral proteins were then assessed using the VaxiJen v2.0 server (https://www.ddg-pharmfac.net). The transmembrane topology of the proteins was evaluated using the TMHMM-2.0 server (https://services.healthtech.dtu.dk). The physicochemical properties of the proteins, including the isoelectric point (pI), half-life, and the GRAVY (Grand Average of Hydropathy) value, which indicates the hydrophilicity or hydrophobicity of the target protein, were analyzed using the ExPASy ProtParam server. The ERRAT score and Z-score graphs were validated using the ProSA-web server (https://prosa.services.came.sbg.ac.at/prosa.php).

### 2.2 Liner B cell epitopes and discontinues B cell epitopes prediction

The Immune Epitope Database (IEDB) was utilized for the prediction of antigenic epitopes on HSV-1 gB, gC, gD, gE, gH, and gL proteins. Access to the IEDB was facilitated via the following Uniform Resource Locator (URL): http://tools.iedb.org/bcell/. The prediction methods employed were as follows: Bepipred Linear Epitope Prediction 2.0, Emini Surface Accessibility Prediction, and Kolaskar & Tongaonkar Antigenicity, with all default parameters retained. Furthermore, the ElliPro server (http://tools.iedb.org/ellipro) was utilized to predict continuous and discontinuous B-cell epitopes.

### 2.3 Activation possibility analysis of IFN-γ, IL-4, and IL-10

The IFN epitope server (http://crdd.osdd.net/raghava/IFNepitope) was utilized to ascertain whether the selected epitopes function as IFN-γ inducers. Furthermore, the IL4pred server (https://webs.iiitd.edu.in/raghava/IL4pred) and the IL-10pred server (https://webs.iiitd.edu.in/raghava/il10pred) were utilized to predict the IL-4 and IL-10 inducing potential of the epitopes.

### 2.4 MHC-I epitope prediction

The enhanced neural network method provided by the NetMHC-4.0 server (https://services.healthtech.dtu.dk) was utilized to predict MHC-I class CD8+ cytotoxic T lymphocyte (CTL) epitopes. In this predictive analysis, all epitope lengths were set to 9-mer by default, as nonapeptide epitopes are recognized by HLA class I molecules and are conducive to vaccine development. The prediction encompassed all allelic sites of HLA-A, HLA-B, and HLA-C, with a particular focus on alleles such as HLA A_01:01, HLA A_02:06, HLA A_29:02, HLA B_15:02, HLA B_40:13, HLA C_03:03, and HLA C_07:01.

### 2.5 MHC-II epitope prediction

The prediction of MHC-II restricted CD4+ helper T lymphocyte (HTL) epitopes was conducted using the NetMHCII2.3 server (https://services.healthtech.dtu.dk), with the epitope length set to 15-mer. This server employs artificial neural networks to predict the binding affinity of epitopes to HLA-II molecules. The prediction included all allelic sites of HLA-DR, HLA-DQ, and HLA-DP, such as HLA-DRB1_01:03, HLA-DRB1_01:01, HLA-DRB1_04:01, HLA-DPA10103-DPB10301, HLA-DPA10103-DPB10401, HLA-DQA10101-DQB10501, and HLA-DQA10102-DQB10501.

### 2.6 Selection of optimal epitopes for vaccine formulation

Following the initial epitope prediction, the epitopes with the highest affinity were selected. These epitopes were then evaluated for antigenicity using the VaxiJen v2.0 server (https://www.ddg-pharmfac.net). The transmembrane topology was predicted using the TMHMM2.0 server (https://services.healthtech.dtu.dk), allergenicity was assessed with AllergenFP (https://ddg-pharmfac.net), and toxicity was evaluated using ToxinPred (https://webs.iiitd.edu.in). The epitopes demonstrating high antigenicity, non-toxicity, non-allergenicity, and full conservancy were selected for vaccine construction. These selected epitopes were integrated into the vaccine design along with adjuvants, including PADRE and human beta-defensin, and specific linkers such as EAAAK, AAY, GPGPG, and KK.

### 2.7 Analysis of biophysical and structural properties of the vaccine

The constructed vaccine was analyzed for antigenicity and allergenicity using the VaxiJen v2.0 and AllergenFP servers, respectively, to ensure its safety and efficacy. Solubility in *E. coli* expression systems was evaluated using the Protein-Sol server (https://protein-sol.manchester.ac.uk/). The biophysical properties, including isoelectric point (pI), molecular formula, instability index, solubility, predicted half-life, and GRAVY (Grand Average of Hydropathicity) value, were assessed using the ProtParam tool on the ExPASy server. Secondary structure prediction was conducted using the SOPMA server (https://npsa-prabi.ibcp.fr). Model validation was performed with the PROCHECK server (https://www.ebi.ac.uk) to evaluate the Ramachandran plot, and the SAVESv6.0 server (https://saves.mbi.ucla.edu/) was used to assess the ERRAT score. The Z-score plot was evaluated using the ProSA-web server (https://prosa.services.came.sbg.ac.at/prosa.php/).

### 2.8 Homology check of autoimmune risk

To evaluate the risk of autoimmune reactions of vaccine constructs 1 and 2, the Blastp tool (https://blast.ncbi.nlm.nih.gov/Blast) in the NCI database was used to compare the protein sequences of V1 and V2 with human protein sequences respectively. Based on their similarities, the risk of possible autoimmune reactions was determined.

### 2.9 3D modeling, refinement and validation

The three-dimensional (3D) structure of the vaccine construct was constructed using the AlphaFold3 tool. The tertiary structure of the vaccine construct was then adjusted using the trRosetta server (https://yanglab.qd.sdu.edu.cn/trRosetta) and the AlphaFold Protein Structure Database (https://alphafold.com/). Subsequent optimization of the structure was performed using the GalaxyRefine module available on the GalaxyWEB server (https://usegalaxy.org/). Finally, PyMol software was employed to visualize and analyze the 3D structure, with a focus on identifying the best spatial resolution for the optimal energy conformation.

### 2.10 Disulfide engineering of the vaccine construct

Disulfide engineering was performed using the Disulfide by Design server to investigate the conformational stability of the vaccine protein. During the analysis, the Cα-Cβ-Sγ angles were maintained at the default value of 114.6 ± 10, and the χ3 angles were set to −87° or +97°. Residue pairs with energy values lower than 2.5 Kcal/mol were selected and converted to cysteine residues to form disulfide bonds, thereby constructing mutant proteins with enhanced stability.

### 2.11 Protein-protein docking analysis

Molecular docking analysis was performed to predict the binding affinity and interaction patterns between the vaccine construct and TLR2, TLR3, TLR4, TLR7/8, and TLR9 receptors. The protein structure sequences of the receptors were obtained from the Uniprot protein database (PDB). Subsequent to this, small compound molecules and water molecules were removed from the receptor structures using PyMOL software. The ClusPro 2.0 server (ClusPro 2.0: protein-protein docking) was utilized for molecular docking to calculate the binding affinity between the vaccine construct and the TLR receptors. The complexes with the lowest energy weighted scores and docking efficiency were determined to be the most successful.

### 2.12 Molecular dynamics and MM/PBSA analysis

MD were performed using the Desmond module in Schrodinger 2021–1 for active ingredients with overall better and better free binding energy scores. After loading the protein, the necessary protein pre-processing work was performed in the protein preparation module, including correction of bonding information, the addition of hydrogen atoms, capping with NME and ACE, and removal of water molecules and excess amino acid chains using OPLS4 force field optimization. Protein-ligand complexes were solubilized using SPC explicit water, set up Orthorhombic Buffer of 10Å. Anti-balance ions were added to the protein-ligand complexes to ensure that the simulated system was electrically neutral, and the closest NPT system to the conventional biological experiments was chosen for the kinetic simulations.

The molecular dynamics simulation workflow consists of four steps: minimization, equilibration, heating, and production. First, the heavy atoms of proteins and small molecules are restricted, and the water molecules are subjected to 5000 steps of steepest descent and 5000 steps of a conjugate gradient to minimize energy; then, the tether is slowly heated to 300k within 30 ps. After the heating is completed, the overall equilibrium of the system is reached at 30 ps. Finally, molecular dynamics simulations were performed for 50 ns at NPT with a time step of 4 fs and 12500 frames, after which correlation analysis was performed using the Simulation Interaction Diagram module. The thermal_mmgbsa.py was used to calculate the binding free energy.

### 2.13 Virtually determine the immune response

The immunogenicity of the recombinant protein and the corresponding immune response curves were analyzed using the C-ImmSim server (https://kraken.iac.rm.cnr.it/C-ImmSim). A FASTA file containing the amino acid sequence was used and the simulation was initiated with the following initial parameters: random seed = 12,345, simulation volume = 10, and simulation steps = 100.

## 3 Results

### 3.1 Selection of potential epitopes from viral glycoproteins

It is acknowledged that viral glycoproteins exhibit antigenic properties and possess desirable physicochemical characteristics. In light of these considerations, the HSV-1 glycoproteins, including gB (UniProt: P06436), gC (UniProt: P10228), gD (UniProt: Q69091), gH (UniProt: Q9DHD5) and gL (UniProt: P10185), were selected for their physicochemical properties analysis (**Supplementary Table 1**), and the identification of non-toxic and non-allergenic epitopes.

To select possible CTL epitopes, the top 25 potential CTL epitopes were identified from the gB, gC, gD, gH, and gL proteins by using NetMHC4.0 (**Supplementary Table 2**). These CTL epitopes are characterized by the presence of extracellular transmembrane domains, a feature that facilitates their recognition. In addition, the CTL epitopes exhibit high binding affinities, as indicated by low binding affinity constants, and antigenicity scores significantly exceed the standard threshold of 0.4, demonstrating strong immunogenic potential.

In addition, the top 25 potential HTL epitopes were selected from viral glycoproteins (**Supplementary Table 3**). These HTL epitopes were found to have binding affinity constants below 100 nM and immunogenicity scores exceeding 1, indicating robust antigen affinity and immunogenicity. Furthermore, the HTL epitopes are conserved, with extracellular transmembrane domains that enhance their accessibility for immune recognition. Finally, nine promising B-cell epitopes were identified based on B-cell epitope evaluations (**Supplementary Table 4**). These B-cell epitopes possess extracellular transmembrane domains and have an immunogenicity score above 0.4, indicating moderate antigenicity.

### 3.2 Multi-epitope based vaccine construction

The identification of the potential CTL, HTL and B-cell epitopes, listed in the **Supplementary Tables 2**-**4**, was undertaken to determine the inducibility of the cytokines (IFN-γ, IL-4 and IL-10). The results demonstrated that at least one of the three cytokines was inducible (**Supplementary Table 5**). A rigorous selection process, adhering to vaccine design standards, yielded 12 CTL, 12 HTL, and 6 B-cell epitopes. The construction of the vaccine was further refined by the strategic incorporation of specific linkers and adjuvants (**Figure 1A**).

In the design of recombinant vaccines, the incorporation of appropriate adjuvants is crucial for enhancing immune responses. This is due to the fact that epitope-based peptide vaccines have been observed to elicit relatively weak immunogenicity when used in isolation (26). β-defensin possesses a multitude of immunological activities, in addition to its direct antimicrobial properties. It also functions as a chemotactic agent that enhances and modulates adaptive immune responses (27). Vaccines incorporating defensins as adjuvants have been shown to activate primary innate antiviral immune responses both *in vitro* and *in vivo*, mediating additional immunomodulatory activities against a variety of viruses (28–30). PADRE, a pan-DR epitope, is a universal helper T-cell epitope capable of activating helper T cells, which in turn assist B-cell antibody production and promote cytotoxic T-cell responses, thereby enhancing both humoral and cellular immunity (31, 32). In this study, the linkers EAAAK, AAY, GPGPG, and KK were used to provide structural flexibility between the fused epitopes, contributing to the stability of the recomb222inant protein and promoting proper antigen folding and exposure for effective immune recognition (33–35). AAY linkers were employed to connect MHC-I epitopes, GPGPG linkers were used for MHC-II epitopes, and KK linkers were utilized for B-cell epitopes. The adjuvants HβD-3 and PADRE sequences were linked using EAAAK linkers at the C-terminal site. Consequently, two candidate vaccine constructs (V1 and V2) were designed, and their amino acid sequences are presented in **Figures 1B, C**.

### 3.3 Biophysical and structural properties analysis of vaccine constructs

Evaluation of vaccine constructs V1 and V2 revealed that construct V1 has a theoretical isoelectric point (pI) of 9.62, an aliphatic index of 91.40, an instability index of 26.73, and a GRAVY value of 0.161 (**Supplementary Table 6**). Construct V2 shows a theoretical pI of 8.17, an aliphatic index of 79.13, an instability index of 33.05, and a GRAVY value of -0.136. Secondary structure analysis of constructs V1 and V2 indicates that construct V1 comprises 20% Alpha Helix, 4.40% Beta Turn, 34.4% Extended Strand, and 41.20% Random Coil, while construct V2 includes 20.53% Alpha Helix, 6.08% Beta Turn, 30.8% Extended Strand, and 42.59% Random Coil (**Supplementary Table 7**).

**Figure 2:**
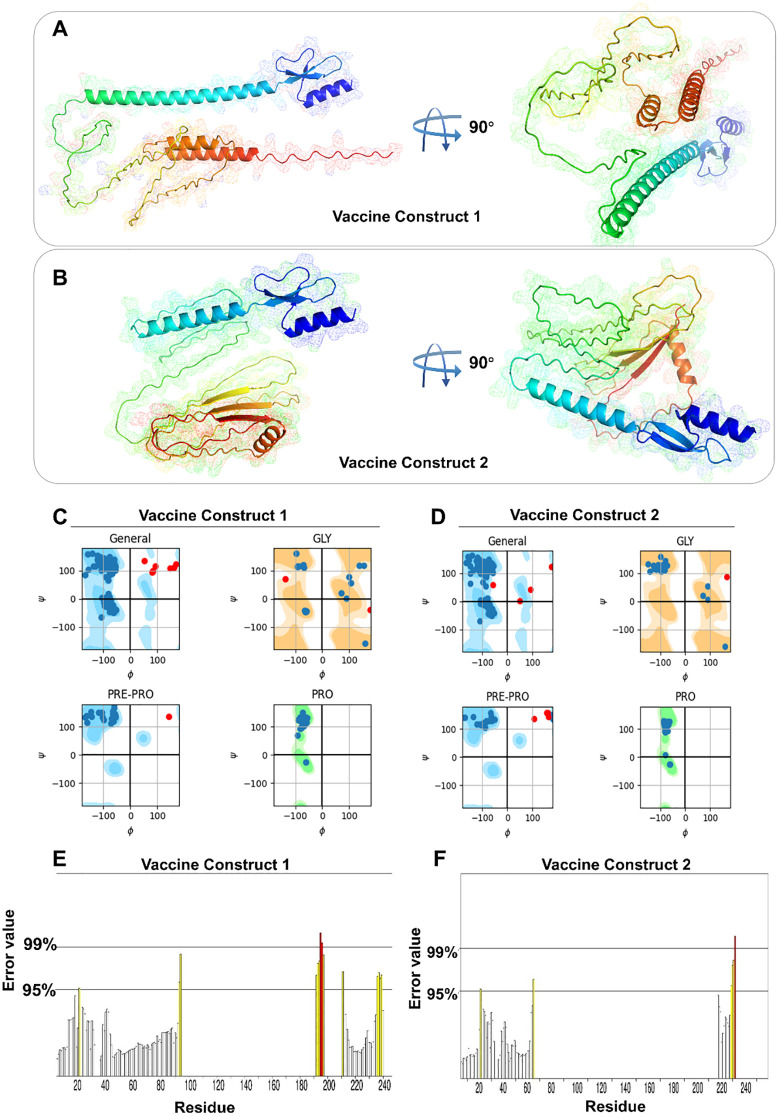
Spatial structure and structural characteristics analysis of vaccine constructs. **(A, B)** 3D model of V1 and V2. **(C, D)** Ramachandran diagram of V1 and V2. **(E, F)** ERRAT mass value of V1 and V2.

Three-dimensional structures of both vaccine constructs were generated, optimized, and validated to yield the most suitable models (**Figures 2A, B**). Subsequent Ramachandran plot analysis of construct V1 revealed that 164 amino acid residues (84.1%) fall within the most favorable regions, 23 residues (11.8%) within allowed regions, 6 residues (3.1%) in less favorable regions, and 2 residues (1.0%) in unfavorable regions (**Figure 2C**). For construct V2, 187 residues (91.7%) are found within the most favorable regions, 15 residues (7.4%) in allowed regions, and 2 residues (1%) in less favorable regions (**Figure 2D**). This analysis indicates that the majority of residues occupy favorable regions, suggesting high stereochemical quality and structural stability, characteristics that are consistent with those of a high-quality protein structure. In addition, the ERRAT scores for V1 and V2 are 88.5246 and 91.5493, respectively, with corresponding Z-scores of -2.17 and -3.17 (**Figures 2E, F**). These high ERRAT scores further indicate that the predicted protein structure has high confidence and accuracy.

### 3.4 Disulfide engineering of vaccine constructs

The construction of mutant proteins is illustrated in **Supplementary Figures S1A, B**. Vaccine construct V1 contains nine pairs of amino acid residues with the potential to form disulfide bonds, while construct V2 has ten such pairs. For both constructs, three residue pairs with energy levels below 2.5 kcal/mol were selected to establish disulfide bonds: CYS11-CYS40, CYS18-CYS33, and CYS23-CYS41.

### 3.5 Assessment of autoimmune risk of vaccine constructs

Following a comparison of V1 and V2 with human protein sequences using the Blastp tool in the NCBI database, it was found that, with the exception of a very high similarity with the adjuvant β-defensin used in the process of vaccine construction (**Supplementary Table 8**), V1 had low similarity with other proteins. In addition to exhibiting a comparatively elevated degree of similarity with the adjuvant β-defensin, V2 also demonstrated a comparatively elevated degree of similarity with Chain A of glycoprotein D (**Supplementary Table 9**). The high degree of similarity between V2 and “Chain A of glycoprotein” may be attributable to the fact that its design itself refers to the glycoprotein sequence of HSV-1. Conversely, it is conceivable that HSV may have evolved this similarity through a process of molecular mimicry in response to prolonged coexistence with the host, thereby evading immune recognition. Despite the presence of structural or sequence overlap, the current data do not provide evidence to suggest that it will directly result in autoimmune complications. Further validation of its immunological safety through animal models or cell experiments is necessary.

### 3.6 Binding affinity of vaccine constructs to TLRs

**Figure 3:**
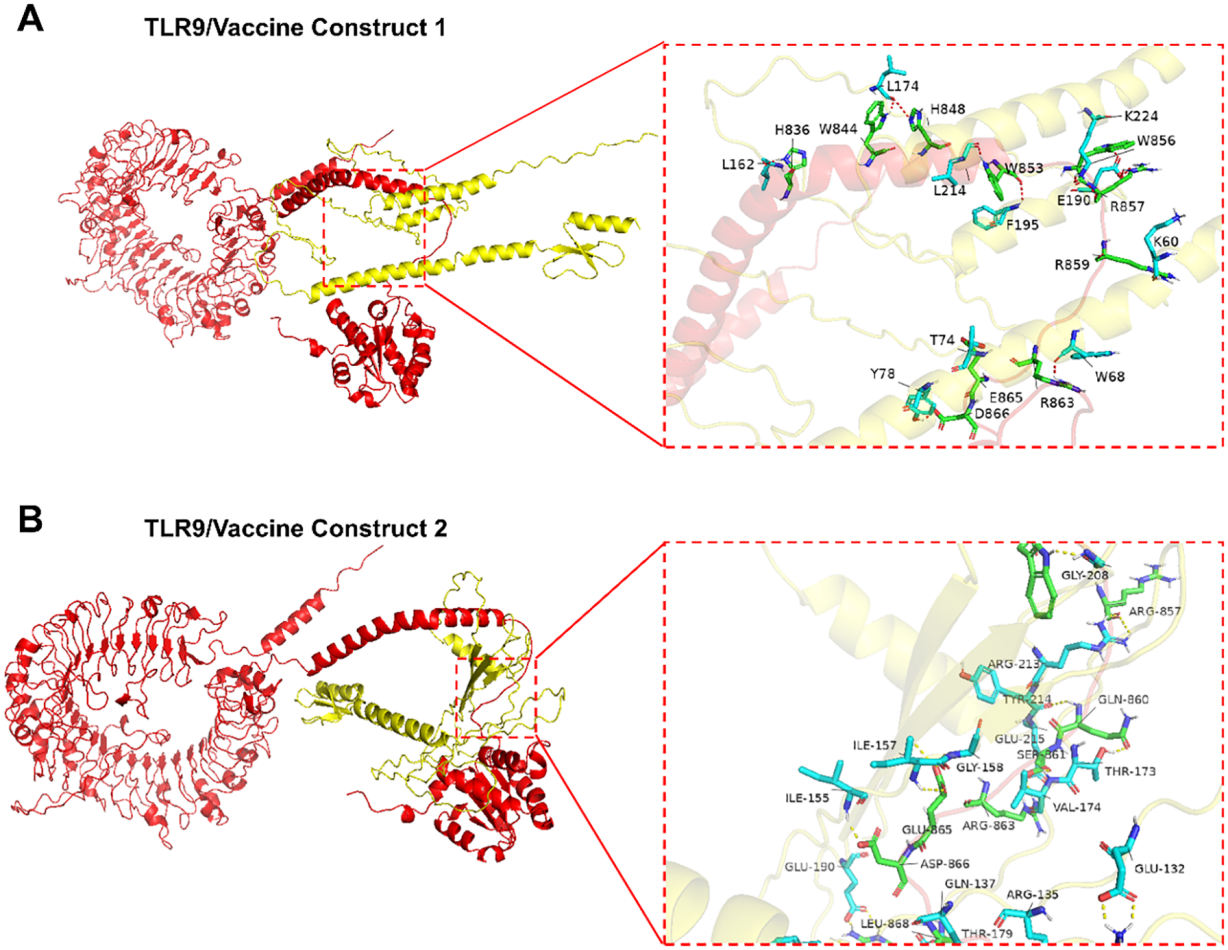
Interactions between TLR9 and vaccine constructs. **(A)** Interaction between vaccine construct 1 and TLR9. **(B)** Interaction between vaccine construct 2 and TLR9.

As the activation of TLRs is critical to stimulate immune responses, molecular docking analyses were performed to evaluate their affinities to the TLR2, TLR3, TLR4, TLR7 and TLR9 receptors. Vaccine construct 1 exhibited a strong binding affinity with TLR9, achieving a docking score of -2298.6 (**Table 1**). Specific amino acid residues in V1, such as LYS60, TRP68, THR74, and TYR78, formed hydrogen bonds and interactions with residues ARG859, ARG863, GLU865, and ASP866 in TLR9 (**Figure 3A**). In contrast, vaccine construct 2 demonstrated even higher binding affinity with TLR9, achieving a docking score of -2418.1. Residues GLN137, ILE155, ILE157, and THR173 in V2 interacted with the active site residues LEU868, ASP866, GLU865, and GLN860 of TLR9 through specific interactions such as hydrogen bonding and van der Waals forces (**Figure 3B**). Therefore, both vaccine constructs exhibit higher affinity to TLR9.

**Table 1:** Binding affinity between vaccine constructs and TLRs.

TLRs	Vaccine 1 Docking Score (kcal/mol)	Vaccine 2 Docking Score (kcal/mol)
TLR2	-1422.1	-1392.4
TLR3	-1515.6	-1693.7
TLR4	-1304.6	-1243.9
TLR7	-1851.7	-1972.8
TLR9	-2298.6	-2418.1

### 3.7 Dynamics interactions between vaccine constructs and TLR9

**Figure 4:**
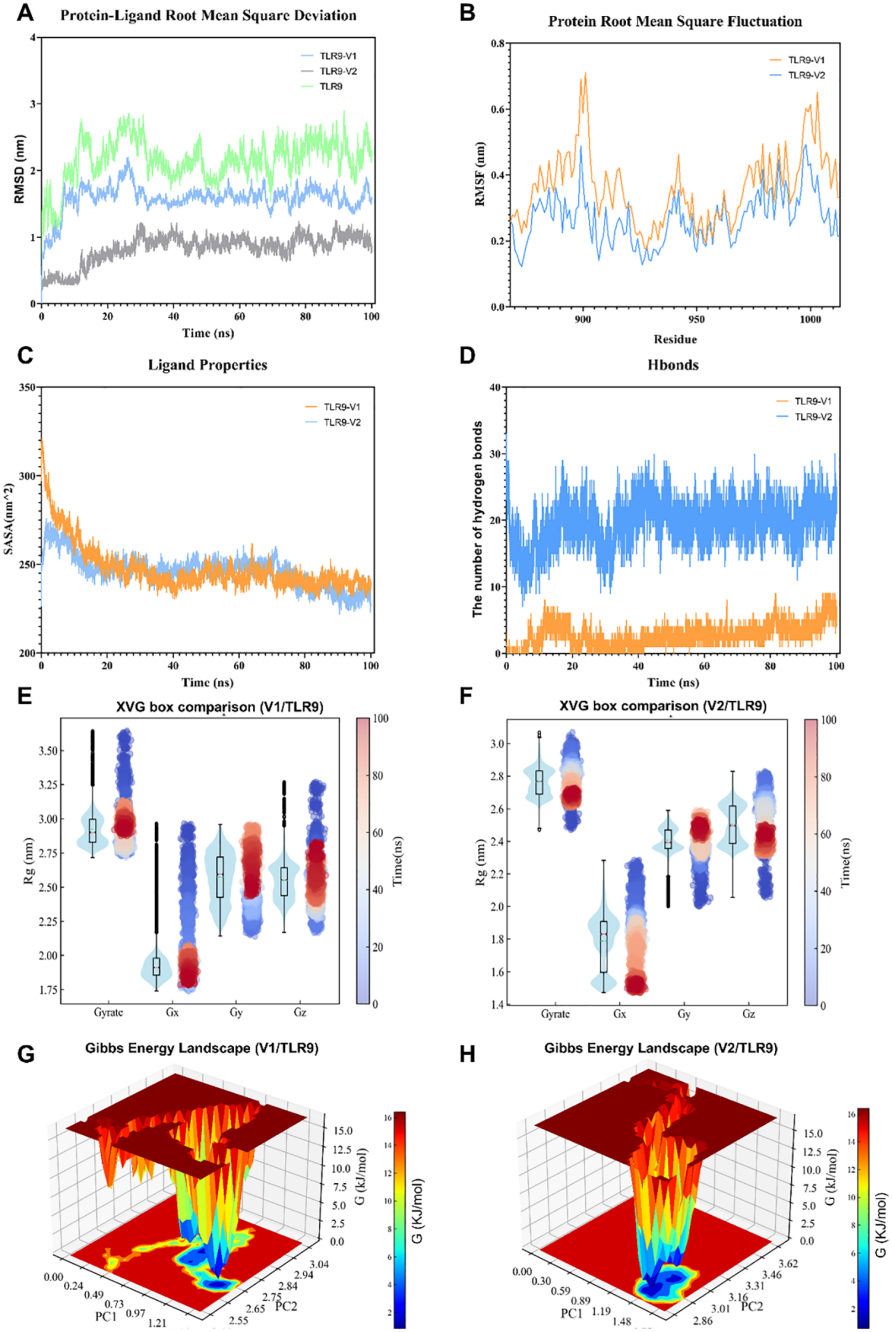
The molecular dynamics simulation of vaccine constructs and TLR9. **(A)** Root mean square deviation analysis of TLR9, the TLR9-V1 complex, and the TLR9-V2 complex. **(B)** Root mean square fluctuation analysis of TLR9 bound to V1 and V2. **(C)** Solvent-accessible surface area analysis of the TLR9-V1 and TLR9-V2 complex. **(D)** Hydrogen bond analysis of the TLR9-V1 and TLR9-V2 interaction. **(E, F)** Radius of gyration (Rg) analysis of the TLR9-V1 complex **(E)** and the TLR9-V2 complex **(F)**. **(G, H)** Binding free energy analysis of the TLR9-V1 **(G)** and the TLR9-V2 **(H)** interaction.

Molecular dynamics simulations were further performed to confirm the interaction between the vaccine constructs and TLR9, with conformational stability and molecular dynamics trajectories of V1 and TLR9 assessed by root mean square deviation (RMSD). In simulations of the TLR9-V1 complex, the structure showed a rapid increase in RMSD within the first 15 ns, followed by stabilization at ~1.5 nm over the next 75 ns (**Figure 4A**). In addition, the root mean square fluctuation (RMSF) values of the V1-TLR9 complex were comparable to those of TLR9 alone, indicating that V1 binding did not significantly interfere with the amino acid residues of TLR9 (**Figure 4B**). Solvent accessible surface area (SASA) analysis demonstrated that the SASA value of the V1-TLR9 complex stabilized at a low level after 40 ns, suggesting that the early conformational change may be related to V1 binding to TLR9 (**Figure 4C**). Hydrogen bond contact analysis showed a continuous increase in the number of hydrogen bonds between TLR9 and V1 over time (**Figure 4D**). Radius changes and orientation distributions indicate that the complex is less stable at the beginning of the simulation, but the overall conformational stability increases with time (**Figure 4E**). Gibbs free energy analysis reveals the presence of multiple low-energy regions within the V1-TLR9 complex, representing a relatively stable conformational state (**Figure 4G**).

In a manner similar to that observed in the V1-TLR9 complex, the RMSD analysis demonstrated that the V2-TLR9 complex stabilized after 25 ns, maintaining values in the around 0.7 nm range (**Figure 4A**). The RMSF values were also found to be similar to those of TLR9 alone (**Figure 4B**). The number of hydrogen bonds between TLR9 and V2 remained steady at approximately 20 (**Figure 4D**). Furthermore, the radius of gyration analysis for V2-TLR9 indicated that the complex was initially unstable; however, its conformational stability improved over time in all directions, aligning with the RMSD trend (**Figure 4F**). The Gibbs free energy analysis of the V2-TLR9 complex over the 100 ns simulation suggested that the system primarily maintained a few stable binding conformations, characterized by low Gibbs free energy values (**Figure 4H**). Conversely, high-energy regions were identified, suggesting the presence of potential conformational barriers that may constrain broader conformational transitions. These barriers must be overcome for structural dissociation to occur, allowing for possible shifts in complex configuration.

### 3.8 Key residues affecting the binding between vaccine constructs and TLR9

**Figure 5:**
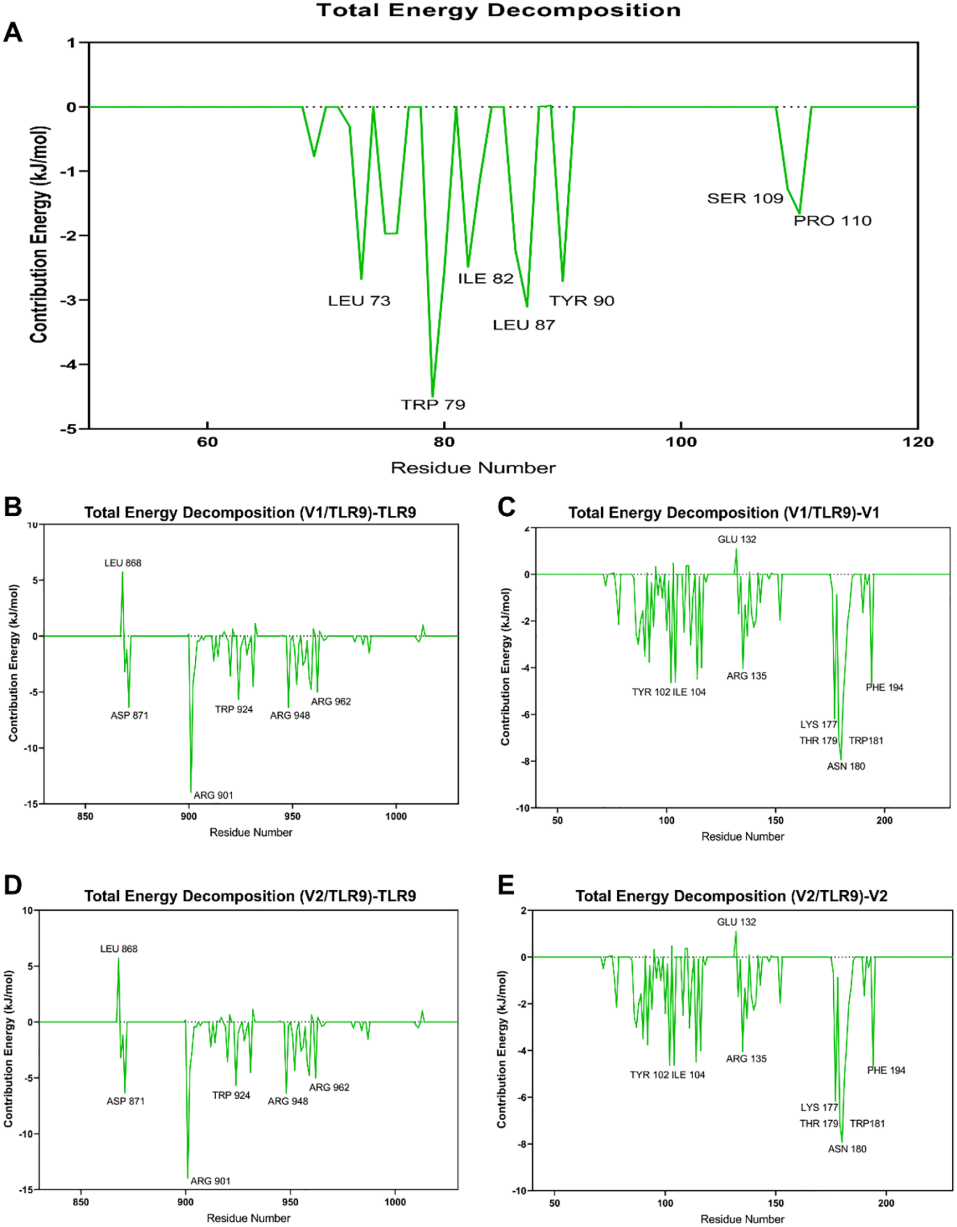
Key amino acids between TLR9 and vaccine constructs. **(A)** The binding energy between the vaccine constructs and TLR9 is evaluated by MM/PBSA Analysis, with different colors indicating various types of binding energy. **(B)** Key residues of TLR9 binding to the V1. **(C)** Key residues of V1 binding to TLR9. **(D)** Key residues of TLR9 binding to the V2. **(E)** Key residues of V2 binding to TLR9.

To clearly decipher the interaction between vaccine constructs and TLR9, the MM-PBSA analysis was performed. In accordance with the data presented in **Table 1**, V2-TLR9 exhibits the lowest total binding energy (-190.60 kJ/mol), signifying the most robust binding, followed by V1-TLR9 (-46.96 kJ/mol). Further analysis revealed that van der Waals forces and electrostatic energy also significantly contribute to the stabilization of the complexes. For instance, the van der Waals energy of the V1-TLR9 and V2-TLR9 complexes is -73.83 and -245.73, respectively, while the electrostatic energy measures 389.13 and -380.35 (**Figure 5A**). This finding provides a molecular-level explanation for the observation that V1 exhibits a weaker binding energy with TLR9.

A more transparent perspective on the interactions within the complexes is offered by dissecting the binding energy by individual residues. The findings indicate that residues TPR-924, TYR928, and LEU959 on TLR9 (**Figure 5B**), along with TPR79, LEU87, and LEU73 on V1 (**Figures 5C**), are pivotal in their binding. However, LEU868 on TLR9 has been shown to have a detrimental effect on their interaction. With regard to the V2-TLR9 binding, residues such as ARG901, ASP871, and ARG948 on TLR9 (**Figure 5D**), along with LYS177, LHR179, ASN180, and TPR180 on V2 (**Figure 5E**), have a positive influence on their interaction. Conversely, certain residues, including GLU132 on V2 and LEU868 on TLR9, have a detrimental effect on their binding.

### 3.9 In silico trial immune simulation

**Figure 6:**
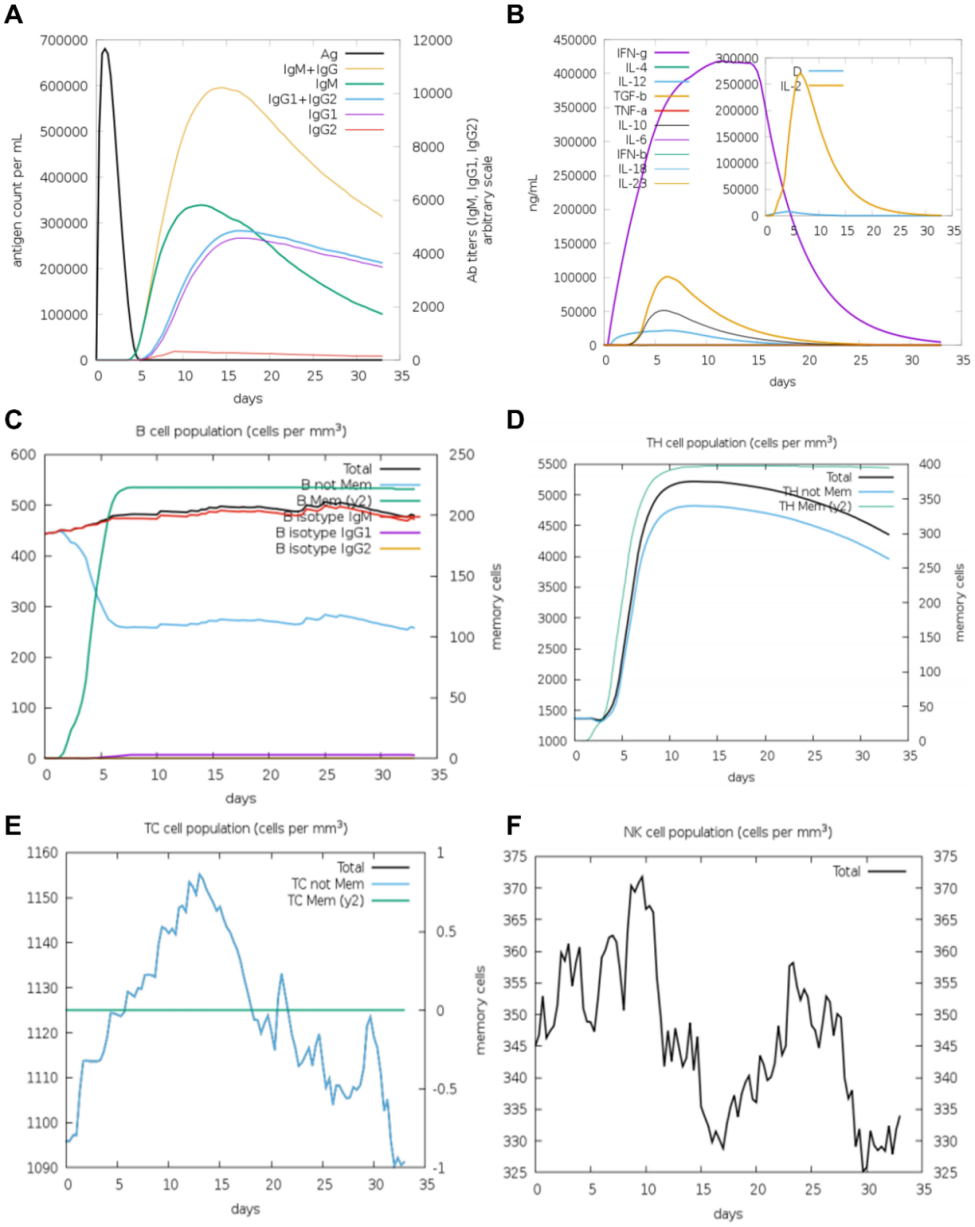
The immune system′s reaction to a simulated vaccine construct 1. **(A)** Response of antigen and immunoglobulines. **(B)** Cytokine response patterns. The ‘D’ in the insert plot represents the danger signal. **(C)** Total count of B lymphocytes and its different subtypes, including memory cells, IgM-, IgG1- and IgG2- isotypes, were shown. **(D)** CD4 T-helper lymphocytes count. **(E)** Total and memory CD8 T-cytotoxic lymphocytes. **(F)** Natural Killer cells.

Finally, we analyzed the immune responses induced by V1 and V2 in silico by using the C-ImmSim platform. Notably, V1 and V2 exhibited distinct characteristics in cytokine profiles, B cell dynamics, and antibody isotype production, reflecting their differential impacts on the host immune system. In the case of V1, IgM remained the dominant isotype throughout the observation period, with minimal contributions from IgG1 and IgG2 (**Figure 6A**). In addition, a comparable cytokine response pattern was elicited by V1, with IFN-γ peaking sharply on day 5 and gradually declining thereafter, signifying its critical role in mediating early pro-inflammatory responses. In a similar manner, IL-2 displayed an early peak corresponding to T cell activation and proliferation. In contrast, IL-4 and IL-10 showed delayed, moderate increases, consistent with their roles in regulating Th2 responses and suppressing excessive inflammation (**Figure 6B**). The total B cell population in the V1 responses reached a plateau of approximately 500 cells/mm³ by day 5, and remained stable throughout the observation period. Both antigens were found to stimulate a rapid expansion of memory B cells, which peaked around day 5, accompanied by a corresponding decline in non-memory B cells (**Figure 6C**). Furthermore, V1 exhibited analogous trends in the induction of TH cell and TC cell populations, as well as in the promotion of NK cell proliferation (**Figures 6D–F**).

**Figure 7:**
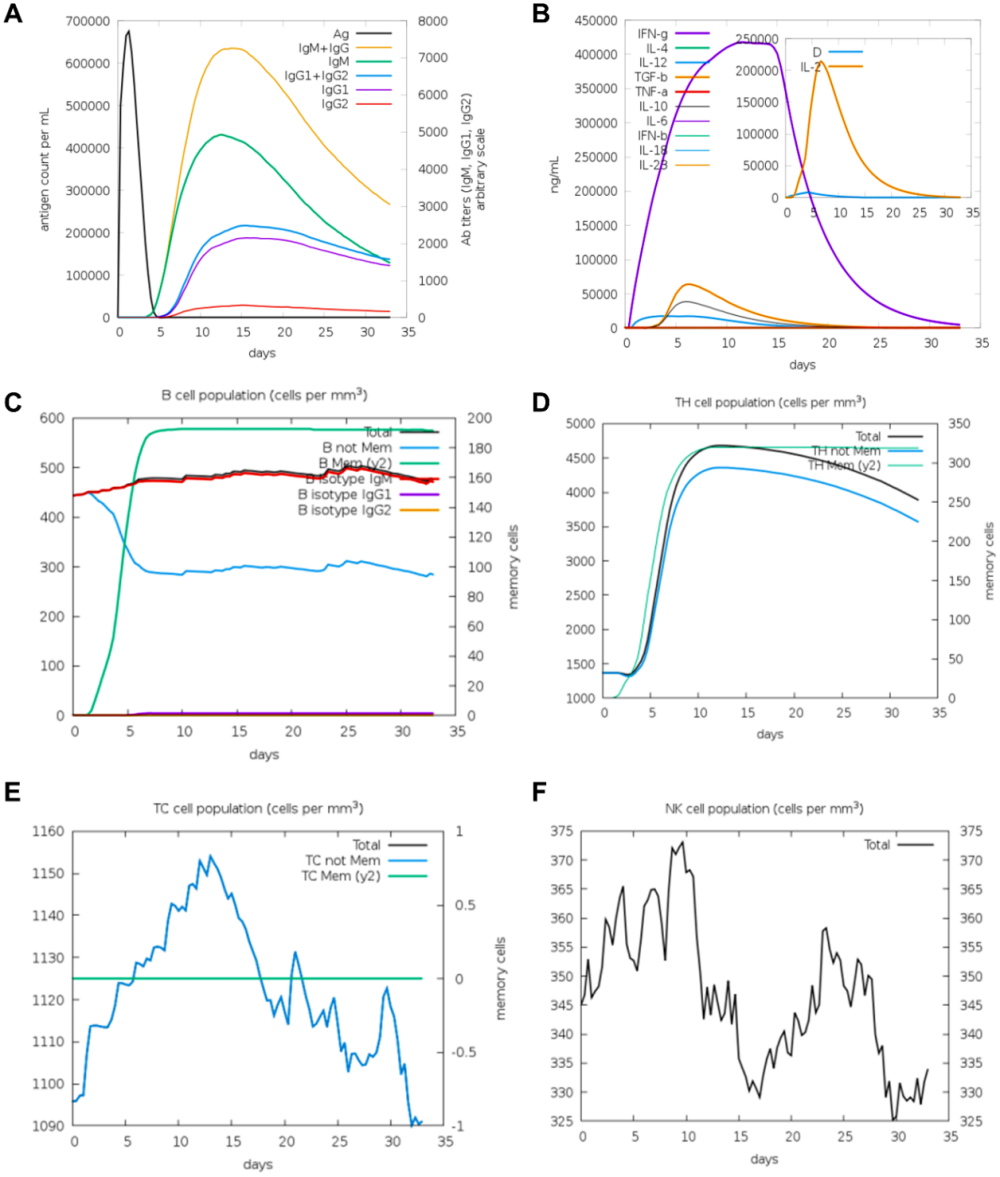
The immune system′s reaction to a simulated vaccine construct 2. **(A)** Response of antigen and immunoglobulines. **(B)** Cytokine response patterns. The ‘D’ in the insert plot represents the danger signal. **(C)** Total count of B lymphocytes and its different subtypes, including memory cells, IgM-, IgG1- and IgG2- isotypes, were shown. **(D)** CD4 T-helper lymphocytes count. **(E)** Total and memory CD8 T-cytotoxic lymphocytes. **(F)** Natural Killer cells.

In contrast, V2 elicited a more diversified antibody response, characterized by slightly lower IgM levels but a detectable increase in IgG1 production, indicative of a shift toward long-term adaptive immunity (**Figure 7A**). V2 also demonstrated superior immunological characteristics by promoting enhanced memory B cell expansion and a shift toward IgG production. In addition, the V2-induced cytokine response exhibited a pattern analogous to that of V1, with a significantly lower level of IL-2 when compared to V1 (**Figure 7B**). This finding may suggest a reduced degree of T cell activation in the V2-induced response. Furthermore, V2 demonstrated a more pronounced and sustained expansion of memory B cells compared to V1, suggesting superior memory formation (**Figure 7C**). Finally, V2 triggered a similar trend in the induction of TH cell, TC cell and NK cell proliferation as V1 (**Figures 7D–F**). Collectively, these observations indicate that V2 may offer enhanced long-term immune protection in comparison to V1, suggesting its potential as a more effective immunological candidate.

## 4 Discussion

In this study, we developed a novel pipeline for designing HSV-1 vaccines, combining bioinformatics and deep learning. Using the UniProt database, we obtained viral protein sequences and constructed two recombinant vaccines targeting five key envelope proteins (gB, gC, gD, gH, gL) from HSV-1 strains. Subsequent bioinformatics analysis demonstrated that these vaccines exhibited both good affinity and immunogenicity, and were non-toxic and non-allergenic to the host. Furthermore, both vaccine constructs met the standards for vaccine design. More importantly, construct V2 exhibits superior immunogenic properties. A greater proportion of amino acids in V2 are located in the most favorable regions of the protein’s three-dimensional structure compared to those in V1, endowing V2 with a more stable tertiary conformation. This structural advantage is likely a key factor contributing to the higher binding affinity score observed between V2 and TLR9, relative to that of V1 and TLR9. In addition, the elevated instability index of V2 in comparison with V1 indicates elevated molecular flexibility. This may, in turn, augment its capacity to elicit host immune responses by means of more efficacious conformational changes.

To date, various types of vaccines have been explored for the treatment of HSV, including replication-defective viral vaccines, naked DNA vaccines, live-attenuated vaccines, trivalent subunit vaccines, nucleoside-modified mRNA vaccines, and viral protein-deleted vaccines. However, their therapeutic efficacy has proven to be suboptimal. The utilization of bioinformatics in the design of novel fusion protein vaccines, comprising multiple single proteins, has emerged as a promising approach to inducing protective immune responses for the treatment of infectious diseases (16). A plethora of strategies are currently being explored for the development of HSV vaccines, though the majority of these strategies rely on conventional methods. A common approach involves the creation of a live attenuated vaccine for HSV, with the aim of inducing lifelong immunity (36). This method involves the genetic modification of the virus to prevent it from causing disease in infected hosts while triggering a broad immune response. However, the process of designing an attenuated live vaccine for HSV is challenging due to the virus’s ability to evade immune surveillance and infect neural tissues. An alternative approach involves the use of DNA-based vaccines, also known as gene vaccines, which are made from plasmid DNA (pDNA) (37). Two primary strategies have been developed for the design of such vaccines: the suppression of viral growth by identifying and destroying cells during the early stages of replication, or the enhancement of the host’s response to HSV surface glycoproteins (gB, gD, or the gH/L complex) using adjuvants or cytokines (16, 38, 39). A representative example is the subunit vaccine, in which a trivalent formulation containing HSV-2 glycoproteins C, D, and E produced via baculovirus expression is administered with CpG/alum as an adjuvant. This vaccine has demonstrated protective efficacy against HSV-2 and cross-protection against HSV-1 (40, 41). Another typical example is the nucleoside-modified mRNA vaccine, which has shown tremendous potential in recent years (41). This platform facilitates rapid immunogen discovery and induces robust immune responses (42). Research has demonstrated that nucleoside-modified mRNA vaccines encoding HSV-2 glycoproteins C, D, and E demonstrate efficacy against both HSV-1 and HSV-2, exhibiting immunogenic effects that are comparable to those of trivalent subunit vaccine immunization (43). The modification of mRNA has been demonstrated to enhance translational efficiency while concomitantly reducing inflammation-associated adverse effects (41). In comparison with the CpG/alum-adjuvanted subunit vaccine, the trivalent nucleoside-modified mRNA vaccine has been shown to demonstrate superior efficacy. However, it is important to note that the efficacy of 9subunit vaccines may be further enhanced through the development of novel adjuvants (43). Nonetheless, these vaccines have limitations, including low protection against HSV, tissue damage at the injection site, and insufficient neutralizing antibody production. Furthermore, mutant viruses created by deleting essential genes required for replication represent an alternative HSV vaccine strategy (44, 45). These viruses are incapable of spreading infection in the host, yet they can still elicit cell-mediated and humoral immune responses. However, the efficacy of such vaccines has been found to be wanting in a number of respects, including the inability to reduce the recurrence of disease, the healing time for genital lesions, and viral shedding rates.

The utilization of bioinformatics and immunoinformatics technologies has garnered significant recognition in the domain of vaccine design and development (46). Bioinformatics tools have the capacity to rapidly identify key targets, a task that would otherwise require years to complete using conventional experimental methods. Furthermore, bioinformatics-based methods facilitate more precise and targeted searches, rapidly providing effective candidate targets, designing antiviral peptides with minimized allergenic potential, and identifying more stable targets that account for viral mutations and variability among strains (47, 48). To date, bioinformatics-driven strategies have yielded significant progress in the field of vaccine development. For instance, during the SARS-CoV-2 pandemic, bioinformatics tools facilitated the rapid identification of conserved regions that fulfilled immunogenic criteria (49). In a similar manner, in the development of a vaccine against Nipah virus (NiV), researchers employed bioinformatics techniques to design the antigenic protein sequence. This resulted in the construction of a chimpanzee adenoviral vector vaccine and a DNA-based vaccine. The immunization of hamsters with these candidates resulted in complete protection against NiV infection. Furthermore, the vaccinated animals exhibited no clinical symptoms (50). Herein, we performed a series of immunogenomic and bioinformatics methods to construct two multi-epitope vaccines (construct 1 and 2) against HSV-1 infection. We also demonstrated that vaccine construct 2 is more suitable for triggering a robust immune response, thus further strengthening the extensive application of bioinformatics-driven strategies for vaccine design.

TLRs play a critical role in the early defense against viral infections (21). They recognize viral components and activate innate immune signaling pathways, inducing IFN-1, pro-inflammatory factors, cytokines, and chemokines (20). To date, TLR2, TLR3, TLR4, TLR7, and TLR9 have been identified as key receptors in recognizing HSV-1 and inducing IFN-1 during viral entry and replication (22, 23). TLR9 typically recognizes unmethylated CpG dinucleotides, leading to the recruitment of the adaptor protein myeloid differentiation primary response 88 (MyD88) through its intracellular domain. MyD88 subsequently recruits and activates the downstream effectors, including TRAF6 and TAK1. TAK1 phosphorylates and activates nuclear factor-κB (NF-κB) to initiate the transcription of a wide array of immune-related genes (51–55). HSV-1 primarily replicates in the oral and epithelial mucosa during the initial phase of infection, and subsequently establishes latency in the trigeminal ganglia (56). TLR9 is predominantly expressed on mucosal epithelial cells, dendritic cells, macrophages, and other immune cells, where it plays a critical role in the antiviral immune response against HSV-1, particularly during the early infection phase and within the trigeminal ganglia. This response is closely associated with the levels of interferon-gamma (IFN-γ) and interleukin-1 (IL-1) (52, 55). Using molecular docking, we predicted the interactions between the two vaccine constructs and the TLR family. Both vaccine constructs V1 and V2 formed relatively stable complexes with TLR9. Molecular dynamics simulations further showed that V2 maintained stability during the simulation and had lower binding energy. This suggests that V2 may activate downstream innate immune signaling pathways by forming a complex with TLR9, thereby providing protection against HSV-1.

Despite the fact that computational simulations have the capacity to rapidly screen antigenic epitopes, evaluate the physicochemical properties and structural stability of vaccine constructs, and perform immune simulations, thus offering valuable insights for vaccine design, there are several challenges that still need to be overcome. A significant challenge pertains to the synthesis of peptide sequences derived from theoretical predictions. In order to ensure that the desired immune response is elicited and that the expressed proteins do not fold in ways that hinder the predicted antibody-antigen binding sites, it is necessary to synthesize and test these peptides in animal models (48). Furthermore, although immunoinformatics analyses have been demonstrated to provide useful information, it should be noted that *in vivo* responses may differ slightly from computational predictions, thus representing a limitation of the current methodology (57). Consequently, the vaccine constructs developed through bioinformatics approaches will be subjected to both *in vitro* and *in vivo* validation in future studies. Specifically, major histocompatibility complex (MHC) binding assays will be conducted to strengthen the predictive findings, and peripheral blood mononuclear cell (PBMC) stimulation assays will be employed to evaluate immunogenicity. Furthermore, an evaluation of antigen expression and cellular toxicity is to be conducted, in conjunction with *in vivo* animal studies, with the objective of ascertaining the safety and protective efficacy of the constructs. Finally, given the mucosal tropism of HSV-1, future studies could explore mucosal delivery routes, such as intranasal or oral administration, to induce local immune responses. The utilization of mucosal adjuvants, including cholera toxin B subunit and chitosan-based systems, may further enhance mucosal immunity and improve the efficacy of the vaccine against HSV-1 at its primary sites of entry (58, 59). Nevertheless, the selection of appropriate bioinformatics and immunoinformatics tools remains instrumental in facilitating the rational design and prediction of more effective prophylactic and therapeutic vaccines.

## 5 Conclusion

The integration of bioinformatics, machine learning, and dynamics simulations has led to the development of a novel vaccine design strategy. Among the constructs, vaccine construct 2 (V2) demonstrates potential as a preventive antibody against HSV-1 infections. This work signifies a progression in the development of HSV-1 vaccines and establishes a basis for future research in this domain.

## Data Availability

The original contributions presented in the study are included in the article/**Supplementary Material**. Further inquiries can be directed to the corresponding authors.
