# Supplementary material for: Computational design and evaluation of multiepitope vaccines against herpes simplex virus type 1
Source: Front Immunol. 2025 Jun 4;16:1581571. doi: 10.3389/fimmu.2025.1581571 (PMC12174051; doi:10.3389/fimmu.2025.1581571)
Supplement: Supplementary file 1 [file Table1.docx]

**Supplementary information**

**Computational design and evaluation of multiepitope vaccines against Herpes Simplex Virus Type 1**

Zibo Zhao, Weixiong Wang, Jiaping Pang, Bei Zhou, Xin Li, Yifei Wang, Kai Zheng, Zhe Ren

**Supplementary figure**

**
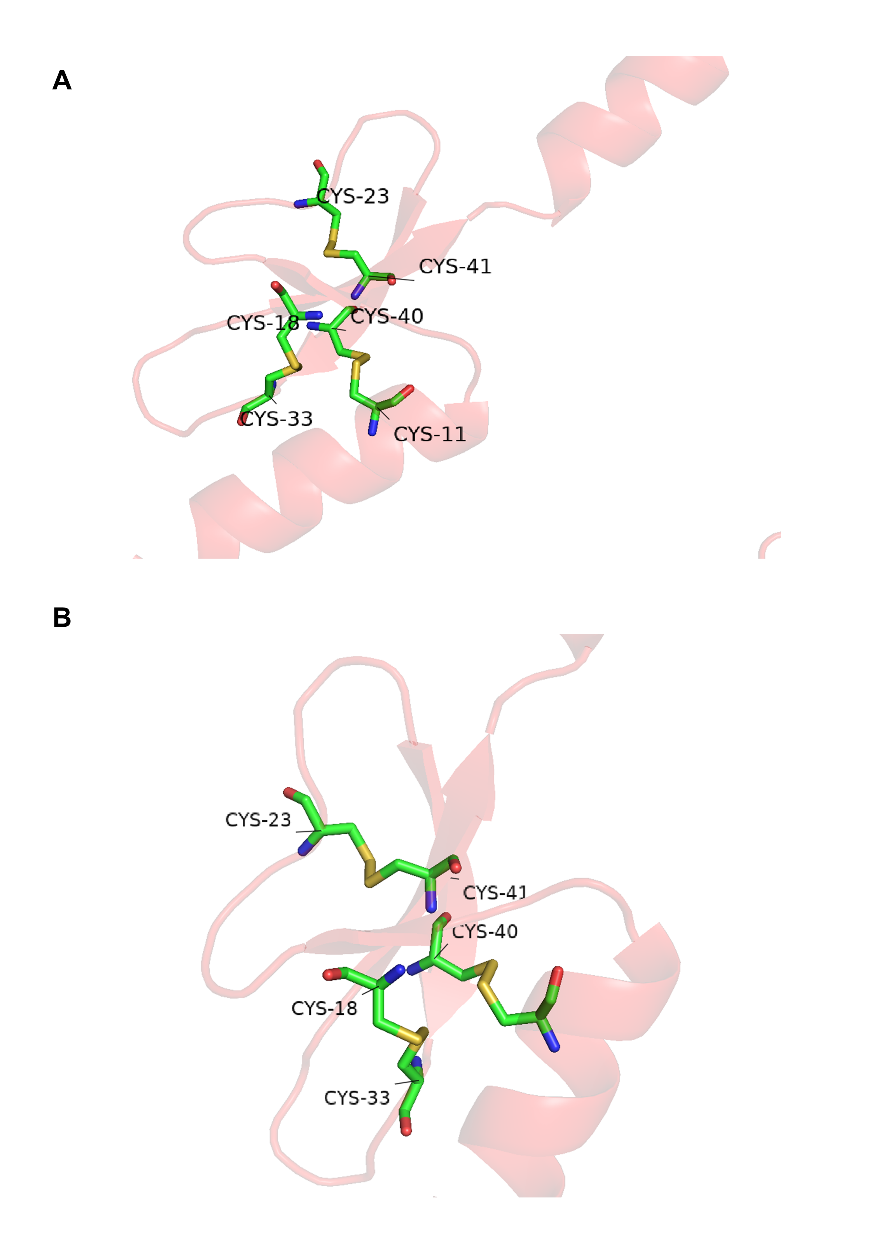
**

**Figure S1. Disulfide engineering of vaccine constructs.** (**A**) Vaccine Construct 1. (**B**) Vaccine Construct 2

**Supplementary Tables**

**Table S1. The antigenicity and physicochemical property analysis of the selected viral proteins.** AN, antigenicity; pI, theoretical pI; AI, aliphatic index; GRAVY, grand average of hydropathicity.

| **Protein name** | **UniProt accession number** | **AN (0.40<)** | **pI (4<)** | **Estimated half life** | **AI (60<)** | **GRAVY** |
| --- | --- | --- | --- | --- | --- | --- |
| Envelope glycoprotein B | P06436 | 0.56 | 7.89 | 30 hours (mammalian reticulocytes, in vitro).  >20 hours (yeast, in vivo).  >10 hours (Escherichia coli, in vivo). | 70.91 | -0.392 |
| Envelope glycoprotein C | P10228 | 0.55 | 7.65 | 30 hours (mammalian reticulocytes, in vitro).  >20 hours (yeast, in vivo).  >10 hours (Escherichia coli, in vivo). | 68.49 | -0.326 |
| Envelope glycoprotein D | Q69091 | 0.54 | 7.64 | 30 hours (mammalian reticulocytes, in vitro).  >20 hours (yeast, in vivo).  >10 hours (Escherichia coli, in vivo). | 89.42 | -0.143 |
| Envelope glycoprotein H | Q9DHD5 | 0.40 | 6.61 | 30 hours (mammalian reticulocytes, in vitro).  >20 hours (yeast, in vivo).  >10 hours (Escherichia coli, in vivo). | 92.28 | 0.047 |
| Envelope glycoprotein L | P10185 | 0.57 | 9.32 | 30 hours (mammalian reticulocytes, in vitro).  >20 hours (yeast, in vivo).  >10 hours (Escherichia coli, in vivo). | 84.46 | -0.355 |

**Table S2. List of the predicted best CTL epitopes and their topology, antigenicity, allergenicity, toxicity, conservancy, and toxicity.** AN, antigenicity; TT, transmembrane topology; AG, allergenicity; CN, conservancy.

| **Protein name** | **Epitope** | | **Affinity(nM)** | | **AN (0.4<)** | | **TT**  **(outside)** | | **AG**  **(Non-Allergen)** | | **Toxicity (Non-toxic)** | | **CN**  **(Con-served)** | |
| --- | --- | --- | --- | --- | --- | --- | --- | --- | --- | --- | --- | --- | --- | --- |
| Envelope glycoprotein B | VWALLGLTL | 95.42 | | 1.4188 | | Outside | | Non-Allergen | | Non-Toxic | | Con-served | |  |
|  | FEDRAPVPF | 36.17 | | 1.284 | | Outside | | Non-Allergen | | Non-Toxic | | Con-served | |  |
|  | AAFFAFRYV | 54.53 | | 1.1531 | | Outside | | Non-Allergen | | Non-Toxic | | Con-served | |  |
|  | LLGLTLGVL | 66.81 | | 1.1263 | | Outside | | Non-Allergen | | Non-Toxic | | Con-served | |  |
|  | LTLGVLVAS | 93.18 | | 1.0195 | | Outside | | Non-Allergen | | Non-Toxic | | Con-served | |  |
| Envelope glycoprotein C | AIEWVGIGI | 109.72 | | 2.5348 | | Outside | | Non-Allergen | | Non-Toxic | | Con-served | |  |
|  | WVGIGIGVL | 55.83 | | 2.1024 | | Outside | | Non-Allergen | | Non-Toxic | | Con-served | |  |
|  | APGRVGLAV | 13.45 | | 1.4298 | | Outside | | Non-Allergen | | Non-Toxic | | Con-served | |  |
|  | GRVGLAVVL | 39.01 | | 1.2304 | | Outside | | Non-Allergen | | Non-Toxic | | Con-served | |  |
|  | STLPISYDY | 36.59 | | 1.0052 | | Outside | | Non-Allergen | | Non-Toxic | | Con-served | |  |
| Envelope glycoprotein D | FQPPSLPIT | 13.69 | | 1.2818 | | Outside | | Non-Allergen | | Non-Toxic | | Con-served | |  |
|  | RLELEDEGV | 57.62 | | 1.1542 | | Outside | | Non-Allergen | | Non-Toxic | | Con-served | |  |
|  | LLPPELSET | 18.91 | | 1.024 | | Outside | | Non-Allergen | | Non-Toxic | | Con-served | |  |
|  | LLPPELSET | 76.72 | | 1.024 | | Outside | | Non-Allergen | | Non-Toxic | | Con-served | |  |
|  | YNPSMGVSV | 89.52 | | 1.0614 | | Outside | | Non-Allergen | | Non-Toxic | | Con-served | |  |
| Envelope glycoprotein H | GRYVYFSPS | 32.08 | | 1.4747 | | Outside | | Non-Allergen | | Non-Toxic | | Con-served | |  |
|  | GIGYKLTGV | 9.55 | | 1.3846 | | Outside | | Non-Allergen | | Non-Toxic | | Con-served | |  |
|  | PLFWRLTGL | 27.39 | | 1.2894 | | Outside | | Non-Allergen | | Non-Toxic | | Con-served | |  |
|  | TEQTDPWFL | 90.84 | | 1.2807 | | Outside | | Non-Allergen | | Non-Toxic | | Con-served | |  |
|  | ELNLTTASL | 87.81 | | 1.2709 | | Outside | | Non-Allergen | | Non-Toxic | | Con-served | |  |
| Envelope glycoprotein L | PSDDLDWRY | 5.52 | | 1.6507 | | Outside | | Non-Allergen | | Non-Toxic | | Con-served | |  |
|  | GLQPKPLTT | 115.3 | | 1.4919 | | Outside | | Non-Allergen | | Non-Toxic | | Con-served | |  |
|  | LIAVGVLCV | 97.69 | | 1.334 | | Outside | | Non-Allergen | | Non-Toxic | | Con-served | |  |
|  | GLIAVGVLC | 103.32 | | 1.1881 | | Outside | | Non-Allergen | | Non-Toxic | | Con-served | |  |
|  | WVGLIAVGV | 33.65 | | 1.0624 | | Outside | | Non-Allergen | | Non-Toxic | | Con-served | |  |

**Table S3.** **List of the predicted best HTL epitopes and their topology, antigenicity, allergenicity, toxicity, conservancy, and toxicity.** AN, antigenicity; TT, transmembrane topology; AG, allergenicity; CN, conservancy.

| Protein name | Epitope | Affinity(nM) | AN  (0.4<) | TT  (outside) | AG  (Non-  Allergen) | Toxicity  (Non-toxic) | CN  (Con-served) |
| --- | --- | --- | --- | --- | --- | --- | --- |
| Envelope glycoprotein B | SIEFARLQFTYNHIQ | 36.2 | 1.4014 | Outside | Non-Allergen | Non-Toxic | Con-served |
|  | SSIEFARLQFTYNHI | 26.3 | 1.2939 | Outside | Non-Allergen | Non-Toxic | Con-served |
|  | DLNITMLEDHEFVPL | 33.8 | 1.5027 | Outside | Non-Allergen | Non-Toxic | Con-served |
|  | LNITMLEDHEFVPLE | 35 | 1.4176 | Outside | Non-Allergen | Non-Toxic | Con-served |
|  | TFIDLNITMLEDHEF | 25.3 | 1.3488 | Outside | Non-Allergen | Non-Toxic | Con-served |
| Envelope glycoprotein C | IEAIEWVGIGIGVLA | 29.1 | 1.73 | Outside | Non-Allergen | Non-Toxic | Con-served |
|  | AIEWVGIGIGVLAAG | 18.8 | 1.6218 | Outside | Non-Allergen | Non-Toxic | Con-served |
|  | VIEAIEWVGIGIGVL | 43.2 | 1.6169 | Outside | Non-Allergen | Non-Toxic | Con-served |
|  | EAIEWVGIGIGVLAA | 22.3 | 1.6122 | Outside | Non-Allergen | Non-Toxic | Con-served |
|  | QVIEAIEWVGIGIGV | 61.5 | 1.5762 | Outside | Non-Allergen | Non-Toxic | Con-served |
| Envelope glycoprotein D | ESQLNLTVMAKPTNW | 30.8 | 1.2138 | Outside | Non-Allergen | Non-Toxic | Con-served |
|  | SANGKPPSVVSWETR | 76.1 | 1.0957 | Outside | Non-Allergen | Non-Toxic | Con-served |
|  | QLNLTVMAKPTNWIE | 18.6 | 1.0504 | Outside | Non-Allergen | Non-Toxic | Con-served |
|  | NLTIAWFRMGGNCAI | 5.1 | 1.0452 | Outside | Non-Allergen | Non-Toxic | Con-served |
|  | SCKYALPLRIPPSAC | 14.9 | 1.4026 | Outside | Non-Allergen | Non-Toxic | Con-served |
| Envelope glycoprotein H | IAPGFLAASALGVVM | 6.9 | 1.0051 | Outside | Non-Allergen | Non-Toxic | Con-served |
|  | YEERFCFVLVTTAEF | 18.4 | 1.5563 | Outside | Non-Allergen | Non-Toxic | Con-served |
|  | EERFCFVLVTTAEFP | 16 | 1.3973 | Outside | Non-Allergen | Non-Toxic | Con-served |
|  | RDLGLVGAVFMRYTP | 19.4 | 1.3737 | Outside | Non-Allergen | Non-Toxic | Con-served |
|  | ELDITHLHNASTTWL | 14.5 | 1.1024 | Outside | Non-Allergen | Non-Toxic | Con-served |
| Envelope glycoprotein L | LGWVGLIAVGVLCVR | 40.4 | 1.96 | Outside | Non-Allergen | Non-Toxic | Con-served |
|  | WVGLIAVGVLCVRGG | 55.3 | 1.7062 | Outside | Non-Allergen | Non-Toxic | Con-served |
|  |  |  |  |  |  |  |  |
|  | GWVGLIAVGVLCVRG | 45.4 | 1.4545 | Outside | Non-Allergen | Non-Toxic | Con-served |
|  | GILGWVGLIAVGVLC | 54.1 | 1.1932 | Outside | Non-Allergen | Non-Toxic | Con-served |
|  | DLDWRYETPSAINYA | 14.7 | 1.7655 | Outside | Non-Allergen | Non-Toxic | Con-served |

**Table S4. List of the predicted B-cell epitopes and their topology, antigenicity, allergenicity, toxicity, conservancy, and toxicity.** AN, antigenicity; TT, transmembrane topology; AG, allergenicity.

| Epitope | Start | End | AN  (0.4<) | TT  (outside) | AG  (Non-Allergen) | Toxicity  (non-toxic) |
| --- | --- | --- | --- | --- | --- | --- |
| SALLSAK | 868 | 874 | 1.2743 | Outside | Non-Allergen | Non-Toxic |
| PPIAPAP | 167 | 173 | 0.9422 | Outside | Non-Allergen | Non-Toxic |
| RVGLAVVLWSLLWLGAG | 5 | 21 | 0.816 | Outside | Non-Allergen | Non-Toxic |
| ELSETPNATQPELAPEDPED | 256 | 275 | 0.7213 | Outside | Non-Allergen | Non-Toxic |
| LGLVGAV | 727 | 733 | 1.3315 | Outside | Non-Allergen | Non-Toxic |
| TTVEPTAQPP | 127 | 136 | 0.9167 | Outside | Non-Allergen | Non-Toxic |
| GWVGLIAVGVLCVRGG | 5 | 20 | 1.7246 | Outside | Non-Allergen | Non-Toxic |
| PCVPLPSDDLDWRYETPSAINY | 43 | 64 | 0.8427 | Outside | Non-Allergen | Non-Toxic |
| TPPPII | 194 | 199 | 0.762 | Outside | Non-Allergen | Non-Toxic |

**Table S5. IFN-γ, IL-4, and IL-10 activation possibility.**

|  | **Epitopes** | **IFN-δ** | **IL-4** | **IL-10** |
| --- | --- | --- | --- | --- |
| CTL | VWALLGLT | Inducer | Inducer | non-inducer |
|  | FEDRAPVPF | Inducer | Inducer | non-inducer |
|  | WVGIGIGVL | Inducer | Inducer | non-inducer |
|  | APGRVGLAV | Inducer | Inducer | non-inducer |
|  | FQPPSLPIT | Inducer | non-inducer | non-inducer |
|  | RLELEDEGV | Inducer | Inducer | non-inducer |
|  | GRYVYFSPS | Inducer | Inducer | non-inducer |
|  | GIGYKLTGV | Inducer | non-inducer | non-inducer |
|  | PSDDLDWRY | non-inducer | Inducer | non-inducer |
|  | GLQPKPLTT | Inducer | Inducer | non-inducer |
| HTL | SIEFARLQFTYNHIQ | non-inducer | Inducer | non-inducer |
|  | SSIEFARLQFTYNHI | non-inducer | Inducer | non-inducer |
|  | IEAIEWVGIGIGVLA | non-inducer | Inducer | non-inducer |
|  | AIEWVGIGIGVLAAG | non-inducer | Inducer | non-inducer |
|  | ESQLNLTVMAKPTNW | Inducer | Inducer | non-inducer |
|  | QLNLTVMAKPTNWIE | Inducer | Inducer | non-inducer |
|  | YEERFCFVLVTTAEF | non-inducer | Inducer | non-inducer |
|  | EERFCFVLVTTAEFP | non-inducer | Inducer | Inducer |
|  | LGWVGLIAVGVLCVR | non-inducer | non-inducer | Inducer |
|  | DLDWRYETPSAINYA | non-inducer | Inducer | non-inducer |
| LBL | SALLSAK |  | non-inducer | non-inducer |
|  | PPIAPAP |  | non-inducer | non-inducer |
|  | ELSETPNATQPELAPEDPED | Inducer | non-inducer | non-inducer |
|  | LGLVGAV |  | Inducer | non-inducer |
|  | GWVGLIAVGVLCVRGG | non-inducer | non-inducer | Inducer |

**Table S6. Antigenicity, Allergenecity, solubility, and biophysical properties of Vaccine construct 1 (V1) and Vaccine construct 2 (V2).**

| **Features** | **V1** | **V2** |
| --- | --- | --- |
| **Antigenicity (0.40<)** | 0.6311 | 0.7052 |
| **Allergenecity** | Non-Allergen | Non-Allergen |
| **Solubility (0.45)** | 0.685 | 0.525 |
| **Number of amino acids** | 250 | 263 |
| **Theoretical Isoelectric**  **point (pI) (4<)** | 9.62 | 8.71 |
| **Formula** | C_1223_H_1908_N_322_O_319_S_9_ | C_1254_H_1958_N_340_O_357_S_9_ |
| **Total number of atoms** | 3781 | 3918 |
| **(Asp + Glu)** | 13 | 25 |
| **(Arg + Lys)** | 29 | 30 |
| **Half-life** | 30 hours (mammalian reticulocytes, in vitro).  >20 hours (yeast, in vivo).  >10 hours (Escherichia coli, in vivo). | 30 hours (mammalian reticulocytes, in vitro).  >20 hours (yeast, in vivo).  >10 hours (Escherichia coli, in vivo). |
| **Aliphatic index (60<)** | 91.40 | 79.13 |
| **Instability index (40>)** | 26.73 | 33.05 |
| **GRAVY** | 0.161 | -0.136 |

**Table S7. Secondary structure analysis of Vaccine Construct 1 (V2) and Vaccine Construct 2 (V2) predicted by SOPMA server.**

| **Parameters** | **V1** | **V2** |
| --- | --- | --- |
| **Alpha Helix** | 20% | 20.53% |
| **Extended Strand** | 34.4% | 30.8% |
| **Beta Turn** | 4.40% | 6.08% |
| **Random Coil** | 41.20% | 42.59% |

**Table S8: Comparison of protein similarity between vaccine construct 1 and host (human) proteins**

| **Protein name** | **Percentage identity** | **Expect value** |
| --- | --- | --- |
| beta-defensin-3 | 97.78% | 1.51E-22 |
| poliovirus receptor-related 1, partial | 35.56% | 0.018 |
| Chain B, Poliovirus receptor-related protein 1 | 35.56% | 0.022 |
| Chain A, Poliovirus receptor-related protein 1 | 35.56% | 0.027 |
| Chain A, Poliovirus receptor-related protein 1 | 35.56% | 0.028 |
| nectin-1 isoform 3 precursor | 35.56% | 0.029 |
| Chain D, Poliovirus receptor-related protein 1 | 35.56% | 0.035 |
| HVEC cell-cell adhesion molecule/herpesvirus receptor, partial | 35.56% | 0.037 |
| nectin-1 isoform 2 precursor | 35.56% | 0.044 |
| nectin cell adhesion molecule 1 | 35.56% | 0.047 |

**Table S9: Comparison of protein similarity between vaccine construct 2 and host (human) proteins**

| Protein name | Percentage identity | Expect value |
| --- | --- | --- |
| beta-defensin-3 | 97.778 | 1.57E-23 |
| Chain A, GLYCOPROTEIN D | 95.238 | 4.30E-04 |
| Chain B, Poliovirus receptor-related protein 1 | 48.214 | 8.34E-04 |
| Chain A, Poliovirus receptor-related protein 1 | 48.214 | 0.001 |
| Chain A, Poliovirus receptor-related protein 1 | 48.214 | 0.001 |
| Chain D, Poliovirus receptor-related protein 1 | 48.214 | 0.001 |
| poliovirus receptor-related 1, partial | 48.214 | 0.001 |
| HVEC cell-cell adhesion molecule/herpesvirus receptor, partial | 48.214 | 0.001 |
| HVEC cell-cell adhesion molecule/herpesvirus receptor, partial | 48.214 | 0.002 |
| nectin-1 isoform 3 precursor | 48.214 | 0.002 |
| nectin-1 isoform 2 precursor | 48.214 | 0.002 |
| nectin cell adhesion molecule 1 | 48.214 | 0.002 |
| poliovirus receptor-related 1 (herpesvirus entry mediator C; nectin), isoform CRA_a | 48.214 | 0.002 |
| nectin-1 isoform 1 precursor | 48.214 | 0.002 |
| nectin 1 | 48.214 | 0.002 |
| poliovirus receptor-related 1 (herpesvirus entry mediator C; nectin), isoform CRA_c | 48.214 | 0.003 |
